# Blood flow restricted walking: does the hypoxic environment compromise walking technique?

**DOI:** 10.3389/fspor.2024.1481315

**Published:** 2025-01-09

**Authors:** Thomas Walden, Nathan Smith, Alasdair Dempsey, Andrew Michael Jonson, Olivier Girard

**Affiliations:** ^1^ The KITE Research Institute, Toronto Rehabilitation Institute, University Health Network, Toronto, ON, Canada; ^2^ Health Sciences, Exercise and Sport Science, University of Notre Dame Australia, Fremantle, WA, Australia; ^3^ School of Allied Health (Exercise Science), Murdoch University, Perth, WA, Australia; ^4^ Centre for Molecular Medicine and Innovative Therapeutics, Murdoch University, Perth, WA, Australia; ^5^ The Department of Health and Biostatistics, Faculty of Health, Arts and Design, Swinburne University of Technology, Melbourne, VIC, Australia; ^6^ School of Human Sciences, University of Western Australia, Perth, WA, Australia

**Keywords:** blood flow restricted exercise, walking, fatigue, kinematics, blood flow restriction (BFR)

## Introduction

Implementing blood flow restriction (BFR) during walking is gaining momentum, with a benefit of this approach being reduced absolute external training intensity, still resulting in chronic adaptations to aerobic fitness (1) and muscular strength (2). BFR-walking leads to acute systemic and local physiological adjustments (3, 4). Systemically, hemodynamic stability is reduced, decreasing venous return, cardiac preload, and stroke volume (5). To compensate, heart rate increases to maintain cardiac output (6) while minute ventilation simultaneously increases as the demand for oxygen rises (2). Locally, reductions in oxygen delivery and removal of metabolic waste create a localized hypoxic environment, lowering muscular pH (3). Consequently, BFR-walking is considered a suitable exercise for load-compromised individuals such as older adults, injured athletes, and patients with chronic musculoskeletal disorders (7).

**Figure 1:**
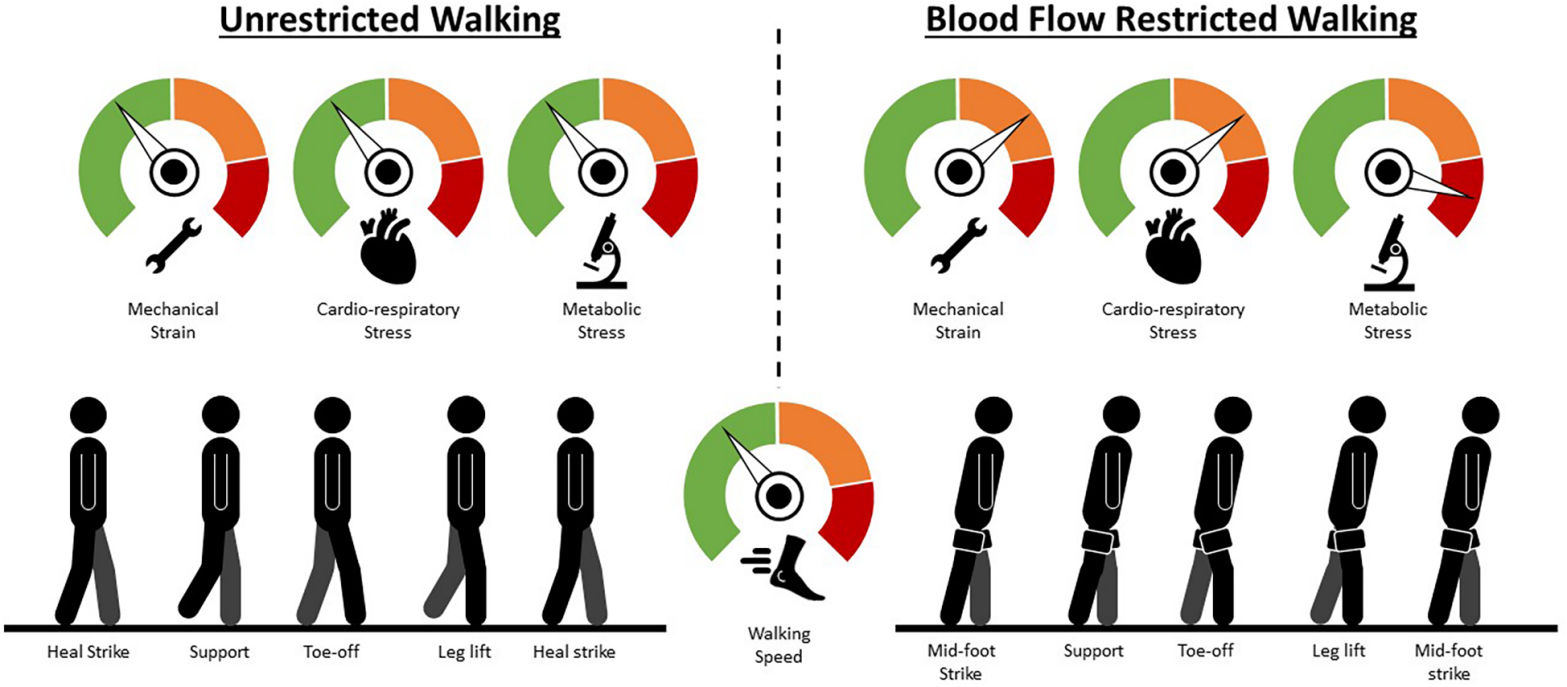
Walking technique changes mediated by blood flow restriction. A hypothetical comparison of the mechanical strain, cardio-respiratory stress and metabolic stress mediated by the application of blood flow restriction and an illustration of the potential compensatory responses an individual may need to employ to maintain walking speed. Green represents low strain/stress; orange represents moderate strain/stress; red represents high strain or stress. Briefly, as blood flow restriction mediates fatigue, force-generation moves from predominantly the ankle to the knee and hip joints (additional knee and hip flexion during the stance phase to increase extension moment of both joints) so that walking speed can be maintained. Simultaneously, the additional knee and hip flexion during the stance phase lowers the height of one's centre of mass and, in conjunction with a wider step width, helps to increase stability.

Literature outside of BFR research indicates acidic environments impair muscle contractility and/or develop a sub-optimal shortening velocity, reducing the power output of muscular contractions (8). Prolonged exposure to the localized hypoxic environment could disturb the contractility of leg muscles during walking. Of particular concern is the reduced contractility of the plantar-flexors, the predominant power generators walking (9). Altered submaximal contractibility of the plantar-flexors could cause neuro-mechanical compensatory strategies and redistribute a portion of the mechanical force created at the ankle to the hip and knee to maintain walking speed (10). To accommodate the redistribution of power, changes in spatio-temporal and/or kinematic characteristics would occur (10). Therefore, we intend to discuss how applying BFR may modify critical characteristics of one's natural gait cycle (Figure 1).

## The influence of muscular fatigue on walking technique

### Power generation

Reduced musculoskeletal function in plantar-flexors requires proximal muscle compensation, redistributing a portion of the mechanical work from the ankle to the knee and hip to maintain walking speed (10). For example, Huang et al. (10) had young adults walk at 5.04 km/h wearing specialized boots bilaterally, setting the maximal plantar-flexion angle and, therefore, artificially mimicking reduced push-off power. A positive relationship was reported between plantar-flexion angle and force production, with the decrease of ankle extension lowering force production. This was compensated by a higher extension moment at the knee to maintain force production (10). Similar changes to the force-generation location were highlighted when comparing younger and older adults (11). The authors reported an age-related reduction in power generation within the trailing leg (plantar-flexors) during level and uphill walking at 4.5 km/h. To compensate, older adults produced greater force during the single-leg support phase, redistributing mechanical power production to the proximal muscles of the knee and hip joints (11).

Additional force generation at the knee and hip joints requires larger muscle groups (*quadriceps, hamstrings*, and *gluteals*) to be activated (12). Larger muscle groups are typically characterized by higher metabolic requirements, increasing the physiological demand of a given task and reducing walking economy (13). To mitigate the higher metabolic cost, individuals may unconsciously reduce their walking speed to a pace they can maintain without altering their walking technique (14). However, if walking speed is constant (common during BFR treadmill walking), neuro-mechanical alterations may occur, causing a faster rate of fatigue development and eventually premature task cessation (15).

### Balance and stability

The body's center of mass during walking is kept predominantly within the base of support between the standing foot and the following contact point of the swinging limb (16). This allows optimal balance and stability, reduces fall risk, and minimizes the cost of locomotion (16). The introduction of fatigue increases inter-stride variability of trunk movement in both the frontal and sagittal planes, termed postural sway (17). In response, stride length and width are altered to maintain balance. For example, Helbostad et al. (16) investigated the impact of fatigue on walking technique among older adults. The fatigue group performed sit-to-stands as quickly and for as long as possible before walking at their preferred speed across the recording area. Participants within said fatigued group exhibited significantly larger step width and slower average walking speed (1.12 km/h fatigued group, 3.56 km/h non-fatigued group), compensatory strategies employed to preserve balance. In addition, step length variability increased, which could represent a compensatory strategy to control anterior/posterior acceleration of the trunk, hence why the study reported little to no postural sway in the sagittal plane.

Compensatory strategies to preserve balance when fatigued during a walking task are not limited to older adults. Nagano et al. (18) assessed walking techniques in young and older adults before and after a 6 min exhausting protocol to identify technique changes related to fatigue. Younger and older individuals reported increased double-support time during preferred speed walking (Older: 3.6 km/h, Younger: 4.21 km/h), another strategy to enhance balance. However, the extended double-limb support time requires increased step length to maintain walking speed (19). Speed maintenance causes increased step width variability, a change widely accepted as an indicator of reduced stability (19). Increased step width can also be detrimental to knee kinematics, exacerbating knee valgus (medial deviation) due to the positioning of the foot relative to the hip (20). As the primary lateral stabilizers of the knee (quadriceps and hamstrings) experience BFR-related fatigue, the gluteals may need to produce additional tension on the iliotibial band, shifting more muscular workload above the occlusion site. These findings highlight a sacrifice in stability when searching for optimal functionality, suggesting this compensatory strategy may not be sustainable across a prolonged walking period. Decreased walking speed and increased step width would inevitably be required to prioritize balance. Overall, balance and optimal functionality are prioritized regardless of age when fatigued.

### Proprioception

Alterations in proprioceptive afferent and efferent signalling can reduce one's ability to sense the location of their limbs (21). Acidosis, ischemic and hypoxic environments directly impact afferent signalling, all elements of muscular fatigue (22). These environmental changes within the muscle reduce the firing frequency of muscle spindles and Golgi tendons, negatively influencing proprioceptive acuity (23). To compensate, afferent inputs from surrounding muscles, joints, vision, and the vestibular system are processed centrally by the cerebellum, so appropriate actions are implemented to maintain balance (21). Once processed, the firing frequency of efferent nerve pathways to the fatiguing limbs increases, activating additional motor units and, therefore, larger muscle fibers (21). A relationship between plantar- and dorsi-flexor muscular fatigue and deficits in postural control has been established (24). The inability to generate small, concise movements at the ankle joint in conjunction with the central processing of proprioceptive signalling reduces the control of one's center of mass, increasing postural sway in both the anterior/posterior and medial/lateral directions (24). The reduction in postural control requires modifications in walking technique to prioritize balance and stability. Additionally, the central processing of proprioceptive information also switches unconscious muscular actions to conscious, increasing the likelihood of a tripping event as obstacle avoidance becomes a dual/secondary task (25).

## The impact of BFR on muscular fatigue

Limited research has investigated neuromuscular function during or following BFR-walking compared to the unrestricted equivalent. However, suggestions can be drawn from low-intensity BFR-resistance exercise research, which has explored how the acute hypoxic environment affects neuromuscular function (26). For example, performing four sets of knee extensions (30% one-repetition maximum) with continuous BFR caused larger reductions in force when compared to without BFR (26). The reduction in muscular performance following all sets suggests BFR mediates additional muscular fatigue distal to the occlusion site compared to the unrestricted equivalent. However, the force decrements within the BFR session dissipated within 2 min post-exercise to the same magnitude of recovery as the unrestricted equivalent post cuff deflation (27). These findings suggest the force decrements stem from the hypoxic environment impairing muscle contractility.

The current prescription guidelines for BFR-walking recommend occlusion be sustained continuously for up to 20 min, which far exceeds the durations (5–10 min) recommended for BFR-resistance exercise (7). Compared to the unrestricted equivalent, decreased muscular force during BFR-resistance exercise was observed less than 1 min into the exercise session (26). The decrements in muscle force continued rapidly as the BFR session progressed (26). The substantial duration of occlusion during BFR-walking could induce a reduction in muscle contractility, similar to BFR-resistance exercise. A decrease in muscle contractibility during BFR-walking would reduce movement speed (14) or reduce exercise time if speed was fixed (15). For example, Sakamaki-Sunaga et al. (15) concluded that BFR led to lower walking speeds (BFR: 4.79 km/h, Unrestricted 6.01 km/h) at which lactate threshold was achieved and reduced exercise time by approximately 12 min compared to the unrestricted group during a graded exercise test. Higher heart rate values were reported during unrestricted walks. Therefore, it could be suggested that the primary reason for task failure was not due to maximal cardiovascular workload. Instead, it may relate to the inability to produce sufficient muscular power to continue.

## Potential impact of BFR on walking technique

We have examined the differences in gait kinematics during BFR and non-BFR walking at both a moderate (5.00 km/h) and fast (6.41 km/h) speed (28). Our main findings were increased knee flexion during stance phase, increased anterior trunk flexion, and increased step width. All findings were exacerbated during the fast condition. These changes could suggest individuals were prioritizing balance during BFR-walking, lowering their center of mass and increasing their base of support, a potential by-product of increased muscular fatigue. The observed increased joint flexion could then be transitioned into larger extension moments at the knee and hip during push-off and single-leg support phases, helping to increase vertical and anterior-posterior ground reaction forces. This is likely a compensatory strategy for a decline in submaximal contractibility of the *gastrocnemius* and *soleus* muscles, reducing the force required from the Achilles tendon during push-off (29), as force generation is redistributed to the knee and hip joints (30).

As the muscles that extend the hip (*gluteal*s) are located above the occlusion site, they initially would not be directly influenced by the hypoxic environment, yet may be relied on more as exercise continues. Increased hip flexion throughout the gait cycle would shift the center of mass forward, potentially outside of one's base of support (12). A rise in stride-to-stride gait instability would result from the movement pattern reorganization, increasing the absolute metabolic cost of exercise (31). An increase in absolute energy requirements in combination with the hypoxic environment could amplify the rate of fatigue due to an intensifying effect (27). Sharp declines in muscle contractility may render the compensatory factors insufficient for some individuals, forcing a reduction of walking speed so that exercise can continue as the mechanical strain imposed on the locomotor system increases. Within our study (28), walking speed was prescribed at a fixed pace based on best practice recommendations. Not all participants were able to finish the prescribed walking time during the BFR sessions. This suggests that at fixed walking speeds, the reductions in contractibility can provoke premature task termination, a finding consistent with Sakamaki-Sunaga and colleagues (15).

## Conclusion

Implementing continuous BFR during walking is associated with exacerbated fatigue and altered gait kinematics, which should be considered in exercise design, particularly for clinical populations. The reported changes in walking technique suggest that further research is required to expand the current BFR exercise guidelines. We suggest altering exercise prescription frameworks and additional monitoring to minimize the risk of falls and/or injury in clinical populations. From the available evidence, prescribing interval-like structures could extend exercise duration by maintaining walking technique and stabilizing the cost of locomotion. Depending on how individuals tolerate the additional fatigue and potential discomfort created by the continuous inflation, cuffs would remain inflated while the exercise stimulus pauses. Leaving the cuffs inflated would offset the rapid neuromuscular recovery observed post-occlusion. Secondly, due to the compensatory alterations associated with BFR, individuals with compromised gait patterns or those recovering from lower limb injuries should be closely supervised. Thirdly, performing the exercise task on a treadmill eliminates external factors (e.g., uneven walking surfaces) that could cause a tripping incident while offering exterior support (e.g., safety rails, harness). Lastly, implementing BFR during alternative exercise modalities (e.g., cycling, rowing, or elliptical) could reduce the risk of injurywhile still offering the physiological benefits of BFR.
